# ASSOCIATIONS BETWEEN INCARCERATION HISTORY AND DIABETES MANAGEMENT AMONG OLDER ADULTS

**DOI:** 10.1093/geroni/igae098.0524

**Published:** 2024-12-31

**Authors:** Sabrina Blank, Rodlescia Sneed

**Affiliations:** Wayne State University, Grosse Pointe Park, Michigan, United States; Wayne State University, Detroit, Michigan, United States

## Abstract

The number of older adults with criminal justice system involvement has increased by more than 280% since 1999. Previous studies show that justice-involved older adults have rates of chronic disease similar to those of the never-incarcerated; however, no studies have evaluated differences in disease management between groups. We examined associations between history of incarceration (HOI) and diabetes management using data from the Health and Retirement Study, a population-based sample of community-dwelling older adults. HOI was assessed via self-report. We assessed diabetes management via self-report/objective health indicators, health service utilization, and health behaviors. We used logistic and linear regression to evaluate associations between HOI and diabetes management, controlling for demographic characteristics. HOI was associated with fewer dental visits (OR: 0.069; 95% CI: 0.531, 0.917) and greater odds of insulin use (OR: 1.52, 95% CI: 1.13, 2.05), tooth loss (OR: 1.94, 95% CI 1.43, 2.65), foot swelling (OR: 1.44, 95% CI: 1.08, 1.90) and hospitalization in the last 2 years, (OR: 1.47, 95% CI: 1.12, 1.92). There was no association between HOI and other measures of diabetes management. Associations between tooth loss, dental visits, and incarceration were observed among White but not Black participants. Findings suggest that HOI may be associated with poorer diabetes management practices in some domains. Future research should identify interventions that improve diabetes-relevant outcomes among justice-involved older adults.

